# Fast high-quality MRI protocol of the lumbar spine with deep learning-based algorithm: an image quality and scanning time comparison with standard protocol

**DOI:** 10.1007/s00256-023-04390-9

**Published:** 2023-06-28

**Authors:** Marta Zerunian, Francesco Pucciarelli, Damiano Caruso, Domenico De Santis, Michela Polici, Benedetta Masci, Ilaria Nacci, Antonella Del Gaudio, Giuseppe Argento, Andrea Redler, Andrea Laghi

**Affiliations:** 1 Department of Medical Surgical Sciences and Translational Medicine, University of Rome “Sapienza” Radiology Unit - Sant’Andrea University Hospital, Via di Grottarossa, 1035-1039, 00189 Rome, Italy; 2 Orthopaedic Unit and Kirk Kilgour Sports Injury Centre, University of Rome “Sapienza” - Sant’Andrea University Hospital, Rome, Italy

**Keywords:** Deep learning, Artificial intelligence, Magnetic resonance imaging, Lumbar spine, Acquisition time

## Abstract

**Objective:**

The objective of this study is to prospectively compare quantitative and subjective image quality, scanning time, and diagnostic confidence between a new deep learning-based reconstruction(DLR) algorithm and standard MRI protocol of lumbar spine.

**Materials and methods:**

Eighty healthy volunteers underwent 1.5T MRI examination of lumbar spine from September 2021 to May 2023. Protocol acquisition comprised sagittal T1- and T2-weighted fast spin echo and short-tau inversion recovery images and axial multislices T2-weighted fast spin echo images. All sequences were acquired with both DLR algorithm and standard protocols. Two radiologists, blinded to the reconstruction technique, performed quantitative and qualitative image quality analysis in consensus reading; diagnostic confidence was also assessed. Quantitative image quality analysis was assessed by calculating signal-to-noise ratio (SNR) and contrast-to-noise ratio (CNR). Qualitative image quality analysis and diagnostic confidence were assessed with a five-point Likert scale. Scanning times were also compared.

**Results:**

DLR SNR was higher in all sequences (all *p*<0.001). CNR of the DLR was superior to conventional dataset only for axial and sagittal T2-weighted fast spin echo images (*p*<0.001). Qualitative analysis showed DLR had higher overall quality in all sequences (all *p*<0.001), with an inter-rater agreement of 0.83 (0.78–0.86).

DLR total protocol scanning time was lower compared to standard protocol (6:26 vs 12:59 min, *p*<0.001).

Diagnostic confidence for DLR algorithm was not inferior to standard protocol.

**Conclusion:**

DLR applied to 1.5T MRI is a feasible method for lumbar spine imaging providing morphologic sequences with higher image quality and similar diagnostic confidence compared with standard protocol, enabling a remarkable time saving (up to 50%).

## Introduction

In primary care, low back pain is among the most frequent reasons for visiting a general practitioner or physiotherapist [1], and low back pain was highlighted as the single highest cause of years lived with disability [2].

Disc degeneration, Modic changes, and facet joint degeneration are most common and possible causes of low back pain [3–5] that can be detected by magnetic resonance imaging (MRI) becoming a commonly used diagnostic imaging modality.

MRI offers many advantages in terms of soft tissue resolution and the absence of ionizing radiation. Nevertheless, it is a method impaired by long acquisition times and relying in parameters’ adjustments in order to improve image quality, eventually resulting in longer scan times [6].

In recent years, artificial intelligence (AI) — in particular deep learning (DL) — is gaining ground in many areas of imaging such as image classification, segmentation, denoising, super-resolution, and image synthesis/transformation [7, 8]. After proper training on a high volume of high-quality images considered as ground truth, DL algorithms allow to reconstruct images with higher signal to noise ratio (SNR), improved spatial resolution, and reduced truncation artefacts, resulting in images with high diagnostic quality and reduced acquisition times compared to standard protocols [9–11].

Among the various DL-based algorithms proposed in recent period, the FDA approved algorithm proposed by GE Healthcare (AIR Recon DL™) uses a deep feed-forward convolutional neural network which reconstructs images with higher SNR, reduces truncation artefacts, and enables higher spatial resolution. Up to now, the pipeline has been trained and optimized on 2D sequences in multiple anatomical regions and for various pulse sequences, contrast weightings, field strengths, and coil configurations [6].

Although many authors have investigated the role of AI application on lumbar spine in different fields such as pathology detection [12, 13] and reporting [14], to the best of our knowledge, the role of AI algorithm on whole routinely MRI lumbar spine protocol acquisition has not been investigated yet.

Thus, the aim of this study is to compare image quality, diagnostic confidence, and scan time between standard protocol and new deep learning reconstruction protocol in lumbar spine MRI.

## Materials and methods

### Patient population

Eighty-five healthy volunteers, with no referred symptoms of lumbar pain, were prospectively recruited from September 2021 to May 2023 and underwent MRI lumbar spine on a 1.5T MR scanner at the Sant'Andrea University Hospital, Rome, Italy. Individuals with incompatible MRI devices, claustrophobic and MRI acquisition with severe motion artifacts were excluded.

This study was IRB-approved and informed consent was obtained by all participants.

### MRI protocol and deep learning image reconstruction (DLR)

**Fig. 1:**
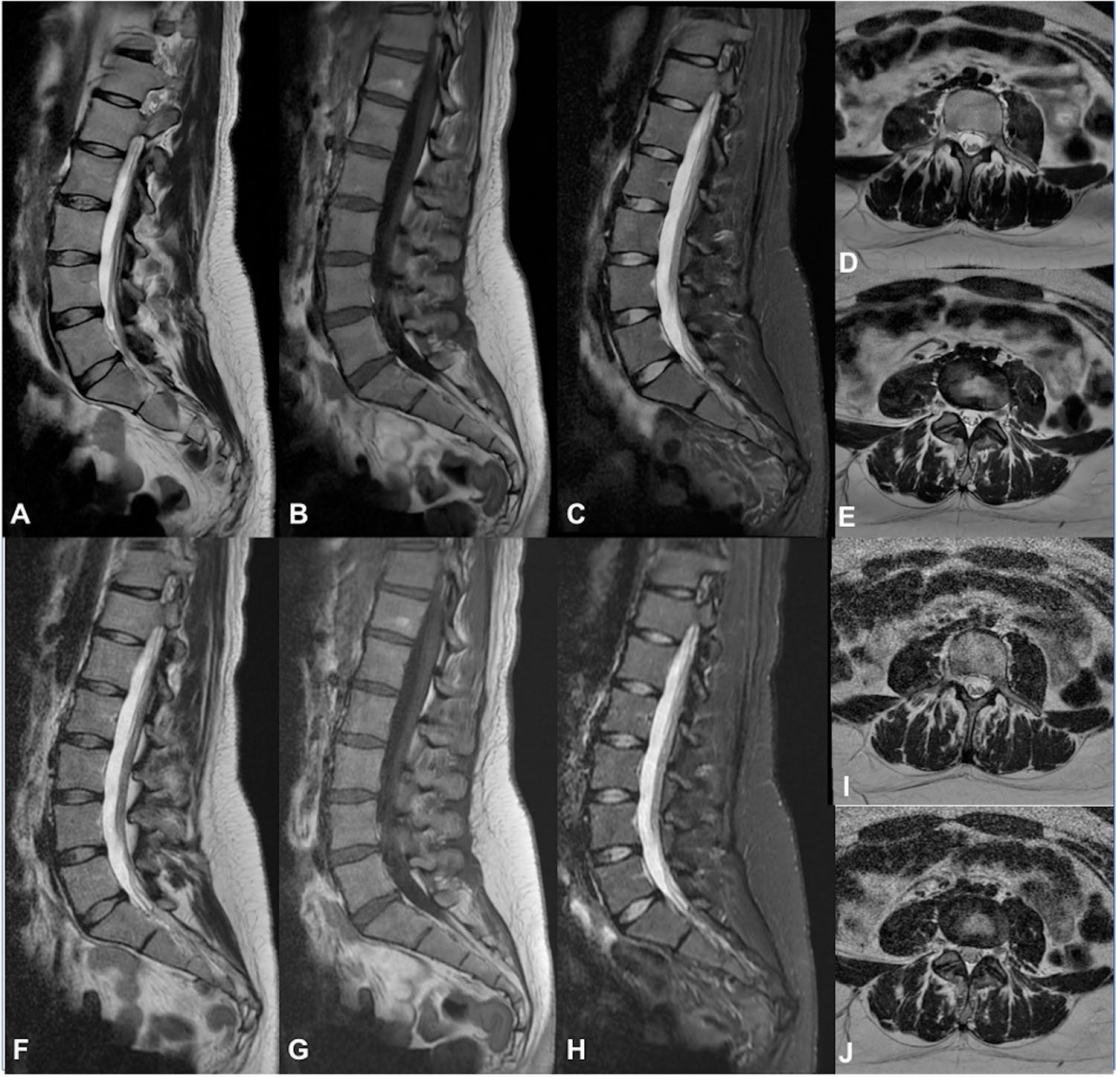
Sequences acquired. On top (**A**–**E**) DLR algorithm. **A**, **D** and **E** T2W FSE; **B** T1W FSE; **C** STIR. Below (**F**–**J**) conventional protocol. **F**, **I** and **J** T2W FSE; **G** T1W FSE; **H** STIR. DLR improves signal-to-noise ratio (SNR), contrast-to-noise ratio (CNR) and image sharpness and reduces artefacts. DLR images appear smoother than standard

All the examinations were performed with a 1.5T MR scanner (Signa Voyager - GE Healthcare, Waukesha, WI) with a dedicated array with 32 coil elements integrated into the patient table (https://integritymed.com/product/ge-signa-voyager-xt-15). Conventional sagittal T1-weighted (T1W) fast spin echo (FSE), T2W FSE, short-tau inversion recovery (STIR) images, and axial multislices T2W FSE images were acquired to compare standard and DLR protocols (Fig. 1). No contrast medium was injected. Acquisition time of each protocol and sequence was collected. Table 1 shows acquisition parameters of both DLR algorithm and standard sequences.

**Table 1:** Acquisition parameters of both DLR algorithm and standard sequences

	DLR				Standard			
	Sagittal			Axial	Sagittal			Axial
	T1W FSE	T2W FSE	STIR	T2W FSE	T1W FSE	T2W FSE	STIR	T2W FSE
Echo time (ms)	7.16	110	55	102	7.76	110	57	102
Repetition time (ms)	467	3230	4149	4848	556	3716	4406	4848
Field of view (mm)	320	320	320	200	320	320	320	200
Matrix	400×320	512×400	384×244	272×192	352×256	416×272	320×192	272×192
Averages	1	1	1	1	2	2	2	2
Bandwidth (Hz)	62.5	62.5	62.5	31.25	31.25	31.25	31.25	31.25
Slices thickness (mm)	3.5	3.5	3.5	3	3.5	3.5	3.5	3
Spacing (mm)	1	1	1	1	1	1	1	1
Slices (*n*)	15	15	15	24	15	15	15	24

AIR Recon DL™ was the algorithm installed on the scanner used to reconstruct images. Its pipeline includes a deep convolutional neural network that reconstructs images with higher SNR, reduced truncation artefacts, and higher spatial resolution. It was trained with a supervised learning approach, using sets of images representing near-perfect and conventional MRI images to generate the high-quality images from low-quality dataset. The software incorporates an adjustable noise reduction factor, ranging between 0 and 1, which represents the fraction of the noise variance to be removed (100% corresponds to removing of all the predicted noise from the image). This pipeline can be applied to 2D sequences in multiple anatomic regions and for various sequences, contrast weightings, field strengths, and coil configurations [6].

### Image analysis

#### Quantitative image analysis

**Fig. 2:**
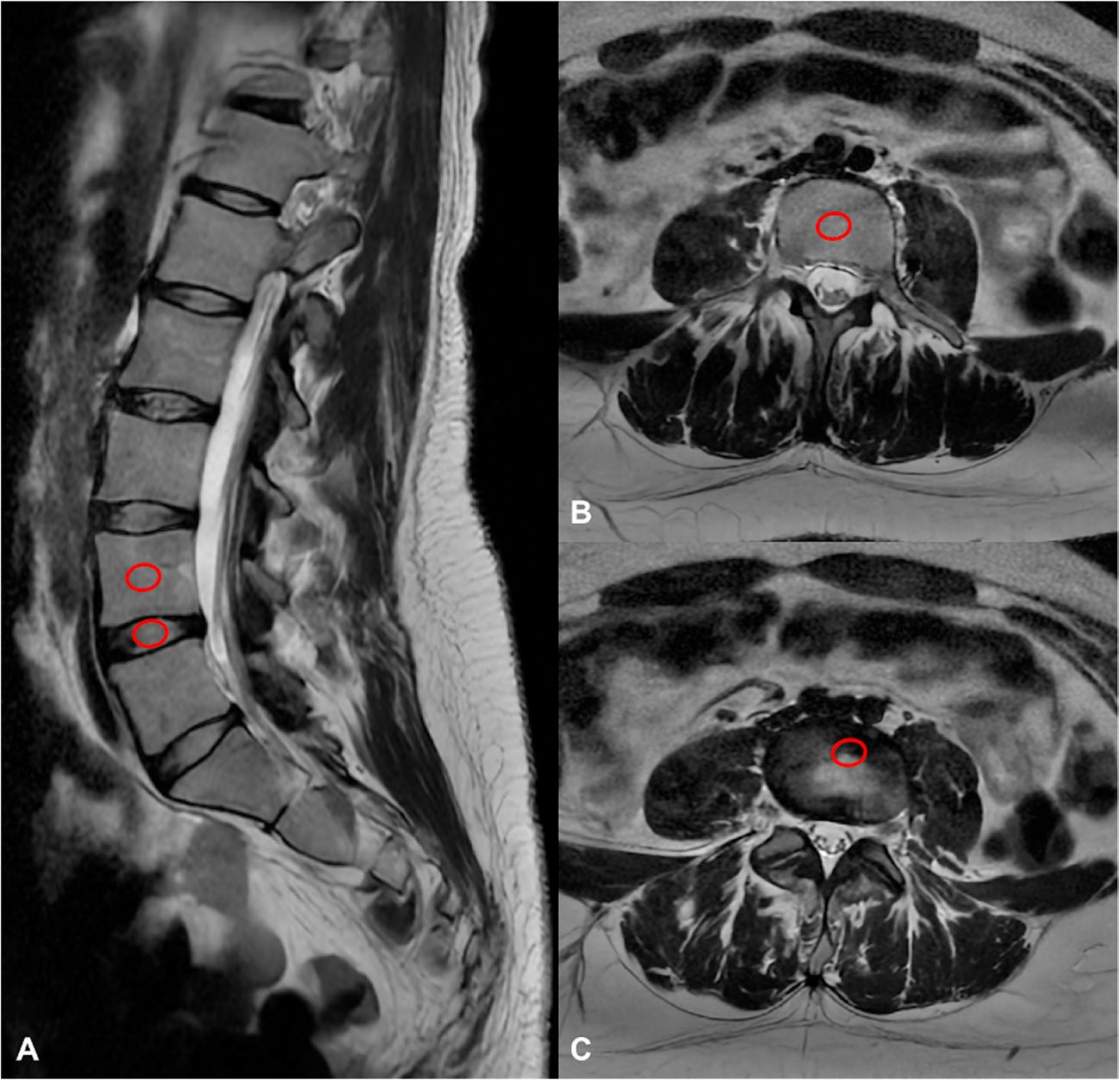
Image analysis process on vertebra and disc of T2W FSE DLR images. **A** Sagittal; **B** and **C** axial images. ROIs are in red. The same analysis was performed on standard protocol images

Two radiologists in consensus (MZ and DDS, with 5 and 7 years of experience in MRI imaging, respectively), blinded to patient details and reconstruction technique, performed quantitative image analysis on a commercially available advanced workstation (AW Server 3.2 Ext. 3.4 - GE Healthcare) by drawing and cloning for each patient/set of images a 10mm^2^ region of interest (ROI) on the fourth lumbar vertebral body (L4) and on the intervertebral disc (L4/L5) in both DLR and standard protocol images (Fig. 2).

Signal intensity, SNR, and CNR were collected. In particular, the average signal intensity of the ROIs was used as the signal intensity, while the standard deviation (SD) was used as the noise. The SNR was calculated for both vertebrae and disc as the signal intensity of tissue divided by the SD of tissue:

$$SNR=\frac{signal\ intensity}{SD}$$

The CNR was calculated as the SNR difference between tissues [15]:

$$\mathrm{CNR}=\left|{\mathrm{SNR}}_{\mathrm{Vertebra}}-{\mathrm{SNR}}_{\mathrm{disc}}\right|.$$

#### Qualitative image analysis

Other two radiologists (FP and DC, with 3 and 10 years of experience in MRI imaging, respectively), blinded to reconstruction technique, independently analysed on a picture archiving and communication system (PACS) all the sequences of both DLR and standard protocol. A five-point Likert scale (5 = excellent; 4 = good; 3 = acceptable; 2 = poor; 1 = non-diagnostic) was used to evaluate the overall image quality.

### Pathologic findings and diagnostic confidence

To avoid recall bias, the two radiologists that performed qualitative analysis, in consensus and still blinded to the reconstruction technique, reanalysed the images after 2 weeks, assessing the presence of pathologic entities including vertebral haemangiomas, Schmorl’s nodes, protrusions, and Modic changes. The analysis included per-patient frequency of pathologic findings and the assessment of diagnostic confidence, using a 5-point scale (1 =non-diagnostic; 2 =poor; 3 =moderate; 4 =good; and 5 =excellent) [16].

### Statistical analysis

Categorical variables are given as median and interquartile range (IQR), continuous variables as mean and standard deviation. Kolmogorov-Smirnov test was performed to establish normality.

Paired *t*-test and Wilcoxon matched paired test were performed to compare data between conventional and DLR images, as appropriate.

Interobserver variability was calculated with the kappa test for the scoring of overall image quality, and it was considered as slight <0.20, fair 0.21–0.40, moderate 0.41–0.60, substantial 0.61–0.80, and almost perfect 0.81–1.00 [17].

Statistical analysis was carried out using MedCalc (MedCalc Software, version15, Ostend, Belgium) with a *p* value < 0.05 that was considered significant.

## Results

### Patient population

**Fig. 3:**
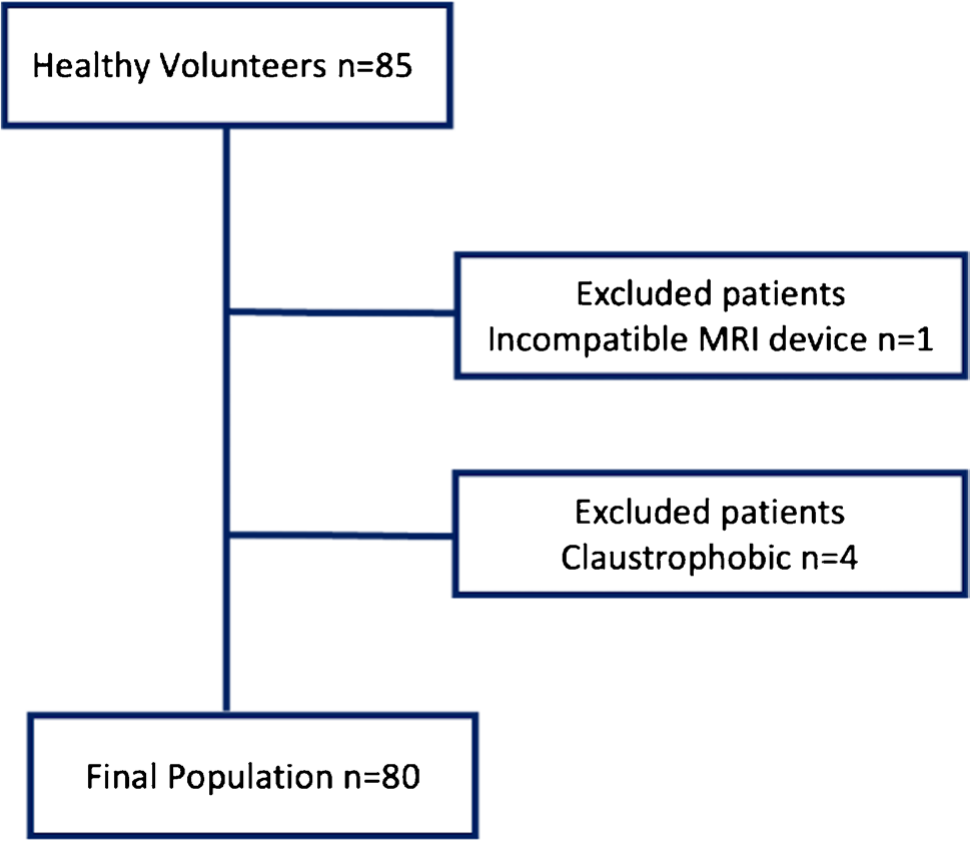
Population enrolment flow chart

From an initial population of 85 healthy volunteers, a total of 80 were included in the study (44 males, 36 females). The participants had a mean age of 42.9 ± 17.1 (range: 21–81) years. Individuals with incompatible MRI devices (*n* = 1 intrauterine device) and claustrophobic subject who requested the interruption of the scanning (*n* = 4) were excluded from the study (Fig. 3).

### MRI protocol and deep learning image reconstruction

Acquisition time was lower for DL-reconstructed images than conventional ones (6:26 ± 0.13 vs 12:59 ± 0.29 min) resulting in a time reduction of 50.2%. In particular, the greatest time reduction was detected for sagittal T1W sequences (106.31 ± 3.51 vs 207.10 ± 4.35 s for DLR and conventional sequence, respectively). Table 2 shows the average times of each sequence.

**Table 2:** Averages time acquisition of both DLR algorithm and standard protocol

	Sagittal			Axial
	T1W FSE	T2W FSE	STIR	T2W FSE
DLR	106.31±3.51	83.46±2.73	104.43±1.43	81.45±1.62
Standard	207.10±4.35	181.10±3.42	188.30±6.53	179.31±19.22
Time reduction	100.79 (48.7%)	97.64 (53.9%)	83.87 (44.5%)	97.86 (54.5%)
*p value*	**< 0.001**	**< 0.001**	**< 0.001**	**< 0.001**

Time reported as average +/− standard deviation in seconds; in brackets time reduction expressed as percentage

Significant *p* values are in bold

### Image analysis

#### Quantitative analysis

The SNR of images reconstructed with DLR algorithm was higher in all sequences compared to standard images (all *p* <0.001).

The CNR of the images reconstructed with DLR algorithm was superior to standard protocol in both T2W FSE sagittal (6.15 ± 7.45 vs 2.20 ± 5.95, *p* < 0.001) and axial images (10.38 ± 4.54 vs 4.91 ± 2.69, *p* < 0.001) whereas no significant differences were reported for T1W FSE (2.67 ± 8.98 vs 4.91 ± 5.37, *p* = 0.170) and STIR images (2.69 ± 7.76 vs 2.25 ± 5.52, *p* = 0.610). Table 3 summarizes SNR and CNR values.

**Table 3:** Signal-to-noise ratio (SNR) and contrast-to-noise ratio (CNR) expressed as mean ± SD of DL algorithm and standard protocol

	T1W FSE			Sagittal			STIR			Axial		
				T2W FSE						T2W FSE		
	SNR		CNR	SNR		CNR	SNR		CNR	SNR		CNR
	Vertebra	Disc		Vertebra	Disc		Vertebra	Disc		Vertebra	Disc	
DLR	17.89±7	15.23±5.62	2.67±8.98	14.85±5.2	8.7±4.9	6.15±7.45	12.67±5.01	9.98±6.59	2.69±7.76	13.98±4.43	3.59±1.62	10.38±4.54
Standard	14.24±4.72	10.15±3.51	4.91±5.37	9.73±3.71	7.52±4.41	2.20±5.95	9.39±4.02	2.41±5.67	2.25±5.52	8.1±2.37	3.19±2.03	4.91±2.69
*p value*	**< 0.0001**	**< 0.0001**	0.1712	**< 0.0001**	**< 0.0001**	**< 0.0001**	**< 0.0001**	**< 0.0001**	0.6165	**< 0.0001**	**< 0.0001**	**< 0.0001**

Significant *p* values are in bold

#### Qualitative analysis

Qualitative analysis showed a greater overall quality in all sequences (all *p* < 0.001) of the images reconstructed with DLR algorithm than standard protocol (median: 4, IQR: 4–5; and median: 5, IQR: 4–5; for reader 1 and reader 2, respectively), with an inter-rater agreement of 0.83 (0.78–0.86). For reader 1, highest quality was found for sagittal STIR (median: 5, IQR: 4–5) and axial T2W FSE (median: 5, IQR: 4–5) and for reader 2 was found for sagittal T1W FSE (median: 5, IQR: 4–5) and T2W FSE (median: 5, IQR: 4–5). Table 4 summarizes scores of the two readers.

**Table 4:** Likert scale scores of the two readers in overall qualitative image analysis

	T1W FSE		Sagittal		STIR		Axial	
			T2W FSE				T2W FSE	
	Reader 1	Reader 2	Reader 1	Reader 2	Reader 1	Reader 2	Reader 1	Reader 2
DLR	4 (4–5)	5 (4–5)	4 (4–5)	5 (4–5)	5 (4-5)	4 (4–5)	5 (4–5)	4 (4–5)
Standard	3 (3–4)	3 (3–4)	4 (3–4)	3 (3–4)	3 (3–3)	3 (2–3)	3 (2–3)	2 (2–3)
*p value*	**< 0.0001**	**< 0.0001**	**< 0.0001**	**< 0.0001**	**< 0.0001**	**< 0.0001**	**< 0.0001**	**< 0.0001**

Data are median (interquartile range)

Significant *p* values are in bold

### Pathologic findings and diagnostic confidence

**Fig. 4:**
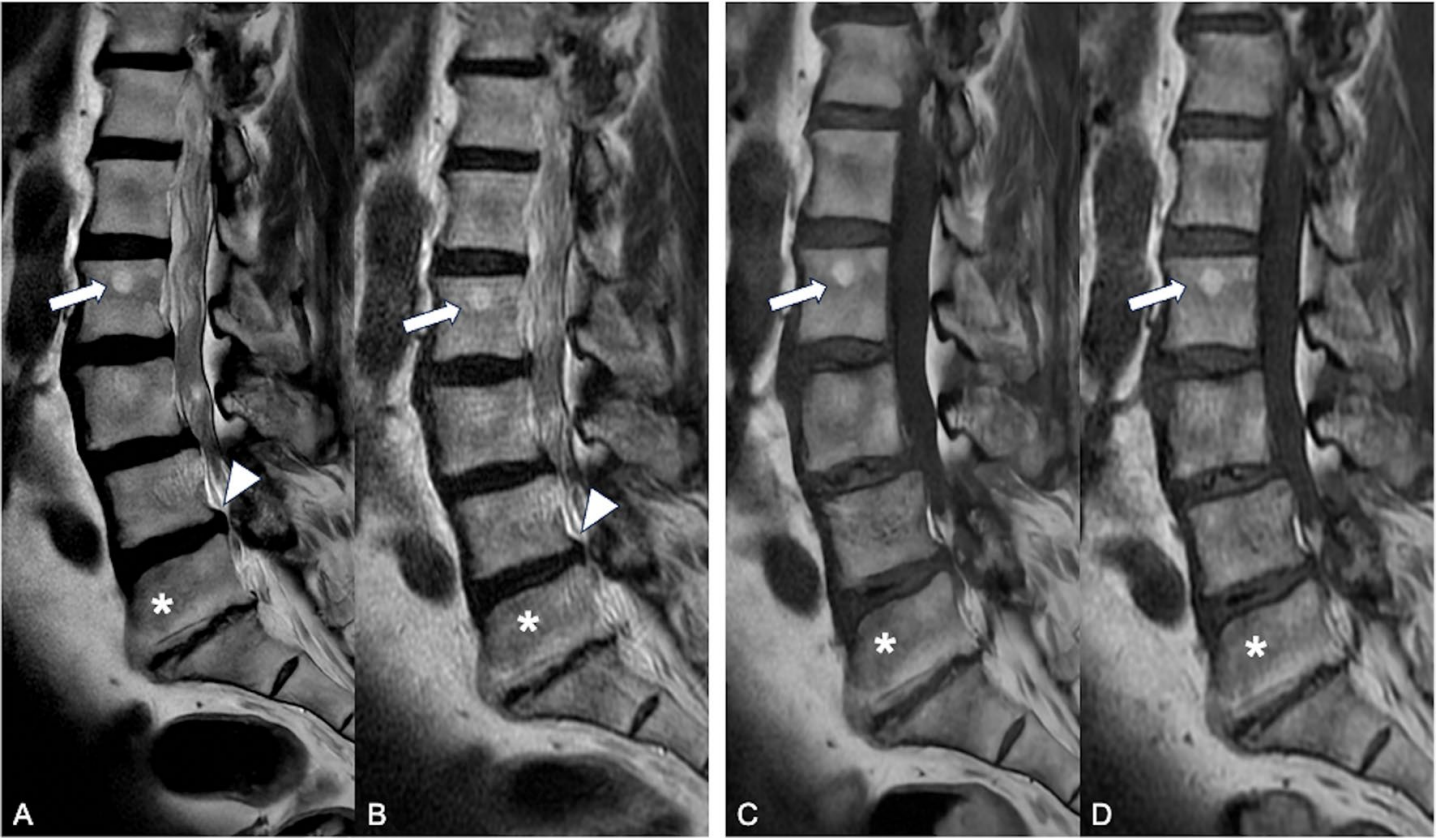
Major pathologic findings detected: haemangiomas (arrows), protrusions (arrows head) and Modic changes (asterisks). **A** and **C** DLR algorithm, T2W FSE. **B** and **D** Conventional protocol T1W FSE

The frequency of pathologic findings did not differ between DLR and standard protocol; findings reported were protrusions (*n* = 11), Modic changes (*n* = 8), haemangioma (*n* = 7), Schmorl’s nodes (*n* = 4) and incidental findings (renal and adnexal cysts, *n*= 4), all *p* > 0.05. Figure 4 and Table 5 summarize pathologic findings reported. None of the findings in both protocols had artefacts (i.e. aliasing or banding artefacts) that might hinder image evaluation.

**Table 5:** The sub-analysis performed on the pathologic findings present on 15 subjects over the 80 subjects included

	DLR	Standard	%
Modic changes	8 (15)	8 (15)	53,3
Protrusions	11 (15)	11 (15)	73
Schmorl’s nodes	4 (15)	4 (15)	26,6
Haemangioma	7 (15)	7 (15)	45,6
Incidental findings	4 (15)	4 (15)	26,6

Numbers and percentage indicate frequency of pathologic findings encountered in both protocols in the 15 subjects included in the sub-analysis

The diagnostic confidence was the same for both DLR algorithm (median: 5, IQR: 4–5) and for standard protocol (median: 5, IQR: 4–5), *p* > 0.05.

## Discussion

Results have confirmed the hypothesis of our prospective study: images acquired with DLR algorithm showed a better quantitative and qualitative image quality and a 50.2% lower scan time than standard protocol on lumbar spine MRI. AIR Recon DL™ may provide a faster scanning protocol for lumbar spine MRI study, providing even a better image quality than the standard protocol. To the best of our knowledge, no one have compared DLR algorithm and standard protocol on routinely MRI lumbar spine exams yet.

In the field of musculoskeletal applications, Herrmann J. et al. [18] evaluated the feasibility of a DL MRI Reconstruction for TSE sequences, comprising lumbar spine. Although the study was performed on a 3.0T scanner of a different vendor, results are in accordance with ours in terms of acquisition time reduction and images qualitative assessment. Nevertheless, authors limited their analysis on sagittal TSE T1W and TSE T2W images and did not perform a quantitative analysis in term of SNR and CNR, while our investigation comprised the whole standard acquisition protocol. We believe that quantitative analysis is an added value as it can allow for a better comparison and can reproducibility among different studies. Additionally, our results are in accordance with a recent investigation [19] focused on DLR in lumbar spine, demonstrating higher image quality, higher diagnostic accuracy, and short acquisition time compared to standard MRI protocol. Of note, the study was performed with a MRI equipment of a different manufacturer than ours, therefore DLR might transversally outperform conventional sequences, regardless the vendor, even if a direct comparative study is still lacking.

Additionally, similar results have been obtained by S. Hahn et al. [20], who compared image quality and the diagnostic performances of standard MRI sequences with accelerated sequences without and with AIR Recon DL™ in the identification of tendons lesions on 3.0T shoulder MRI examinations. Their results showed similar subjective image quality, artefacts, and diagnostic performance with standard sequences, obtaining a 67% scan time reduction using accelerated DLR sequences. The availability of arthroscopy as reference standard was an added value of the investigation; however, the lack of quantitative analysis represented a limitation of the study.

In the field of cardiac imaging Velde N. et al. [21] evaluated the influence of AIR Recon DL™ on late gadolinium enhancement image quality on myocardial scar quantification in patients with suspected or known cardiomyopathy. They demonstrated that DLR improved image quality on visual scoring, SNR, and CNR compared to the standard MRI scans. Although their results are obtained on images acquired on 1.5T MRI scanner after injection of contrast medium, they are in agreement with ours. This leads us to underline the potentiality of DLR application on different anatomical districts. Diagnostic performance of AIR Recon DL™ was also investigated by Kim M. et al. [22] in the evaluation of pituitary gland pathology. Their study was conducted comparing 1-mm slice thickness MRI with DLR with 3-mm slice thickness MRI for identifying residual tumour and cavernous sinus invasion in the evaluation of postoperative pituitary adenoma. Results of both quantitative and qualitative analysis showed that the sensitivity of DLR sequences was higher than that of conventional sequences in the identification of residual tumour and cavernous sinus invasion. Unfortunately, no data of acquisition time was reported. Albeit their analysis was conducted on images with different slice thickness than ours, both quantitative and qualitative results are in accordance with ours. This leads us to assume that the algorithm, regardless of the sequence acquisition parameters, can still improve the image quality in both qualitative and quantitative terms.

Regarding clinical impact of the DLR protocol, several advantages might be routinely achieved. For instance, shorter acquisition time helps to improve patient compliance. As demonstrated by Schreiber-Zinaman J. and colleagues on a different anatomical district [23], multiple sequences in abdominal MRI can result in patient dissatisfaction and inefficient resource utilization. This principle can also be applied in lumbar spine district; in fact, shorter examination time translates to higher patient compliance, especially in claustrophobic individuals or in patients who have severe back pain and difficulty in maintaining supine position for a long time. Another related advantage of reduced scan time is the possibility, in case of diagnostic necessity, to add further sequences without significantly affecting acquisition time, scheduling time and patient discomfort.

In addition, considering the reduction in acquisition time, further consideration about costs can be made. Bratke G. and colleagues [24] showed how scanning time reduction is an effective way to increase cost efficiency with MRI. This DLR algorithm, with a total of 50% of time reduction, can also facilitate departmental cost-optimization; further studies tailored on benefit-costs balance are needed to confirm the impact of such important time reduction.

There were some limitations in our study. First, this was a single-centre study with a small number of participants. Second, we only enrolled asymptomatic individuals. As results, only 15 individuals had incidental lumbar pathologies. Third, we did not investigate the AIR Recon DL™ effect on diagnostic performances in a large variety of spine pathology including (e.g. nerve inflammation or damage, severe discitis, trauma) nor on a large sample size.

In conclusion, AIR Recon DL™ images resulted in higher qualitative and quantitative image quality compared with standard reconstruction protocol, with significant shorter acquisition protocol. Therefore, we suggest that DLR protocol can be safely implemented in clinical practice; further multicentre prospective investigations are needed to investigate its impact on diagnostic accuracy.

## Data Availability

The data and analytic code for this study may be available from the corresponding author on reasonable request.

## Abbreviations

SNR: Signal to noise ratio
CNR: Contrast to noise ratio
MRI: Magnetic resonance imaging
DLR: Deep learning-based reconstruction
STIR: Short-tau inversion recovery
T1W: T1-weighted
T2W: T2-weighted
FSE: Fast spin echo
ROIs: Region of interest
